# Social Brain Hypothesis: Vocal and Gesture Networks of Wild Chimpanzees

**DOI:** 10.3389/fpsyg.2016.01756

**Published:** 2016-11-24

**Authors:** Sam G. B. Roberts, Anna I. Roberts

**Affiliations:** Department of Psychology, University of ChesterChester, UK

**Keywords:** chimpanzee, gestural communication, vocal communication, bonding, social network analysis, social complexity, communicative complexity, proximity

## Abstract

A key driver of brain evolution in primates and humans is the cognitive demands arising from managing social relationships. In primates, grooming plays a key role in maintaining these relationships, but the time that can be devoted to grooming is inherently limited. Communication may act as an additional, more time-efficient bonding mechanism to grooming, but how patterns of communication are related to patterns of sociality is still poorly understood. We used social network analysis to examine the associations between close proximity (duration of time spent within 10 m per hour spent in the same party), grooming, vocal communication, and gestural communication (duration of time and frequency of behavior per hour spent within 10 m) in wild chimpanzees. This study examined hypotheses formulated a priori and the results were not corrected for multiple testing. Chimpanzees had differentiated social relationships, with focal chimpanzees maintaining some level of proximity to almost all group members, but directing gestures at and grooming with a smaller number of preferred social partners. Pairs of chimpanzees that had high levels of close proximity had higher rates of grooming. Importantly, higher rates of gestural communication were also positively associated with levels of proximity, and specifically gestures associated with affiliation (greeting, gesture to mutually groom) were related to proximity. Synchronized low-intensity pant-hoots were also positively related to proximity in pairs of chimpanzees. Further, there were differences in the size of individual chimpanzees' proximity networks—the number of social relationships they maintained with others. Focal chimpanzees with larger proximity networks had a higher rate of both synchronized low- intensity pant-hoots and synchronized high-intensity pant-hoots. These results suggest that in addition to grooming, both gestures and synchronized vocalizations may play key roles in allowing chimpanzees to manage a large and differentiated set of social relationships. Gestures may be important in reducing the aggression arising from being in close proximity to others, allowing for proximity to be maintained for longer and facilitating grooming. Vocalizations may allow chimpanzees to communicate with a larger number of recipients than gestures and the synchronized nature of the pant-hoot calls may facilitate social bonding of more numerous social relationships. As group sizes increased through human evolution, both gestures and synchronized vocalizations may have played important roles in bonding social relationships in a more time-efficient manner than grooming.

## Introduction

Primate sociality is frequently characterized as being especially complex in its nature, and primates have unusually large brains for their body size when compared to other mammals. The “social brain hypothesis” proposes that the complex social world of primates is especially cognitively demanding, and that this imposed intense selection pressure for increasingly large brains (Byrne and Whiten, 1988; Dunbar, 1998). Group size in primates is strongly correlated with brain size, and specifically with neocortex size in relation to the rest of the brain, but exactly what makes larger groups more complex than smaller groups is poorly understood (Dunbar, 2003). The complexity of primate social groups depends on the complexity of individual relationships between animals, because the social system itself is an emergent property of these micro-level interactions (Hinde, 1966). Thus, to understand the complexity of social groups, a detailed understanding of how primates interact with others to build and maintain social relationships over time is required, as this is at the heart of what makes primate life socially complex (Dunbar and Shultz, 2010). Other species also come together in large groups (e.g., grazing ungulates such as wildebeest), but these are aggregations of animals, with less stable group membership and thus less stable social relationships between individuals (Haddadi et al., 2011). In contrast, primates live in groups with stable membership, and form long-lasting bonds with certain individuals within the group, where they flexibly respond to one another in repeated instances of affiliative interaction (Dunbar, 1992b). Individual variation in the nature of these social bonds has direct fitness consequences—for example, the sociality of adult female baboons (as measured by grooming and proximity to others) is positively associated with both their own (Smuts, 1985; Palombit et al., 1997; Silk et al., 2010b) and their offspring's survival (Silk, 2007). It is the dynamic and multi-facetted nature of these social relationships, and the need for individual primates both to keep track of its own relationships, and the relationships of other group members (third party relationships), that is hypothesized to drive the social complexity of primate life (Silk, 1999; Engh et al., 2006; le Roux et al., 2013; Roberts and Roberts, 2015).

Thus, one of the distinctive characteristics of primate sociality is its complexity, with complex social systems defined as those in which individuals communicate frequently in many different contexts with many different individuals, and repeatedly interact with many of the same individuals over time (Freeberg et al., 2012). The fact that the neocortex ratio correlates strongly with typical group size lends support to the idea that the larger neocortex in primates evolved under selection to manipulate information about social relationships. The social brain hypothesis assumes that cognitive processing capacities (represented by relative neocortex size) place an upper limit on the size of groups that can be maintained as a cohesive social unit. Primates do not maintain equally strong relationships with all group members, but form differentiated, stable, long-lasting bonds with both related and unrelated group members (Pepper et al., 1999; Langergraber et al., 2009; Mitani, 2009; Silk et al., 2010a). One of the primary mechanisms that primates use for creating and maintaining social bonds is grooming, which can account for up to 20% of their total daytime activity budget. The amount of time primates spend grooming is positively related to group size, suggesting that when groups are large, primates have to spend more time maintaining their social relationships than in small groups (Aiello and Dunbar, 1993; Lehmann et al., 2007). However, the amount of time primates can devote to grooming is limited, because of the demands of other essential activities, notably feeding, resting, and moving (Dunbar, 1992a). Thus, social bonding in primates is constrained by two independent variables—neocortex size which sets an upper limit to the number of relationships individual primates can keep track of, and the amount of time that is available for grooming, which is necessary to maintain social relationships at a sufficient level to prevent the bond from decaying (Dunbar, 1993; Lehmann et al., 2007). If the number of individuals in a group becomes too large, it becomes increasingly difficult for individuals to maintain social bonds with all group members. Thus, group cohesion will decrease and the bonds will eventually decay. For example, the probability that a baboon group will split increases with increasing group size (Henzi et al., 1997). This seems to be determined not by inefficient foraging in larger groups or by predation risk, but directly by the inability of individuals to service social relationships in the face of the inevitably limited amount of time available for social interaction (Henzi et al., 1997). However, it is increasingly being recognized that in addition to grooming, vocalizations (sounds made with the vocal tract) and gestural communication (voluntary movements of the arm, hand, head, or whole body; Roberts et al., 2014a,b) may also play key roles in developing and maintaining social bonds in primates. Time constraints limit the amount of time available for grooming (Lehmann et al., 2007), but vocal and gestural signals are less constrained by time, and thus may offer an important additional way to regulate social relations in groups of primates. Comparative analysis has demonstrated that evolutionary increases in the size of the vocal repertoire in non-human primates were associated with increases in both group size and also time spent grooming (McComb and Semple, 2005). This suggests that vocal communication may play a role in maintaining groups of primates—larger groups are more complex to manage, and thus require a larger vocal repertoire to maintain an increasing number of differentiated relationships. Further, differences in the amount of time devoted to affiliative gestural communication, but not other types of gestures, across three macaque social systems, provides an indication that gestural communication may be used flexibly to maintain a differentiated set of social relationships (Maestripieri, 2005). However, systematic studies of how vocalizations—and especially gestures—are associated with social relationships in primates are in their infancy, despite the potential significance of such studies for furthering our understanding of social evolution in both primates and humans.

Chimpanzees are an excellent species to examine this question because they have complex social dynamics. In the chimpanzee fission-fusion social system, the association patterns change by means of the fission and fusion of subunits (known as parties or sub-groups) according to both the activity (e.g., resting, feeding) and distribution of resources (Pepper et al., 1999). Individuals thus stay in close proximity with some conspecifics from the wider community at infrequent intervals, often weeks apart, but each individual can recognize members of their own community and is capable of maintaining long-term relationships with these individuals (Boesch, 1996; Barrett et al., 2003; Muller and Mitani, 2005; Amici et al., 2008; Eckhardt et al., 2015). Reciprocated social relationships are a key feature of the chimpanzee social system and are marked by increased time and energy investment in repeated and reciprocated instances of association and interaction (Watts, 2006; Mitani, 2009). Chimpanzees also have social relationships with non-reciprocated social partners or weakly bonded conspecifics with whom they have less frequent association and interaction (Foerster et al., 2015). A recent study showed that the presence of reciprocated close proximity bonds between pairs of chimpanzees (i.e., those pairs who spent larger amounts of time in close proximity, per hour spent in the same party) was associated with several behavioral indices. These included a longer duration of visual attention directed at the dyad partner, a longer duration of mutual grooming and received grooming, and a longer duration of time spent resting and traveling, per hour the pair of chimpanzees spent in close proximity (within 10 m; Roberts and Roberts, 2016). Moreover, chimpanzees use a communication system consisting of gestures (Leavens et al., 2004; Forrester, 2008; Hobaiter and Byrne, 2011; Roberts et al., 2012a,b, 2013, 2014a; Smith and Delgado, 2013; Bard et al., 2014) and vocalizations to maintain their relationships (Van Lawick-Goodall, 1967, 1968; Goodall, 1986; Mitani and Nishida, 1993; Mitani et al., 1999; Roberts and Roberts, 2016). For instance, chimpanzees use visual gestures with strongly bonded individuals and tactile or auditory gestures with weakly bonded individuals (Roberts and Roberts, 2016). Gestural communication that has previously been suggested to be important in relation to social bonds includes gestures made when encountering each other after a natural period of separation, in response to the threat of aggression or after receiving aggression (Roberts et al., 2014a; Taglialatela et al., 2015). Vocal communication hypothesized to be important in relation to social bonding in chimpanzees includes pant-hoot calls produced solo or jointly with group members in conjunction with visual or auditory gestures (Mitani and Nishida, 1993; Fedurek et al., 2013) and one-to-one calls (e.g., low intensity pant-grunt calls produced by a subordinate individual towards a dominant chimpanzee). Whilst it is well-known that chimpanzees use a wide variety of gestures and vocalizations when interacting, there have been no systematic studies of how both vocal and gestural communication relate to association and grooming patterns in chimpanzees.

In this study we predict that the number and strength of close proximity relationships maintained with others (expressed as duration of time spent within 10 m per hour spent in the same party) are associated both with biological factors (e.g., maternal kinship, age similarity, sex similarity, reproductive similarity; Huchard et al., 2016) and social bonding (communication and grooming). Specifically, we hypothesize that grooming and affiliative communication have a bonding function through reducing the risk of aggression and therefore are associated with close proximity. Thus, proximity bonds, grooming, and dominance-aggression gestures will correlate, indicating a cost to sociality. However, when affiliative communication and grooming are included in the model, the relationship between the dominance-aggression gestures and proximity will become weaker. Thus, the bonds chimpanzees will have with other individuals will be differentiated, with strong social relationships based on grooming and affiliative communication, whereas weaker social relationships will be based on dominance communication, as chimpanzees use different types of behavior to maintain the different types of bonds.

In addition to these group level associations between communication and proximity, individual chimpanzees also display a large amount of variation in the size of their individual proximity networks. The size of this network reflects the number of conspecifics with whom individual chimpanzees maintain close proximity. The larger the size of the individual proximity network, the greater the time and cognitive demands on maintaining these more numerous social relationships. Thus, we predict that in smaller networks, chimpanzees will form relatively strong ties with all network members, with frequent interactions based on affiliative communication and grooming behavior (Mitani, 2009). However, as individual network size increases, the ties chimpanzees will have with other individuals will become increasingly weak, with less frequent interactions and an increasing dissociation between strong and weak association networks. These weaker, indirect ties are cognitively complex to manage, and this is especially true in fission-fusion social systems where the frequency of interaction between two individuals will be much lower than in other social systems where there is a greater degree of temporal and spatial cohesion between group members (Barrett et al., 2003).

One manner of communication that could be used to service these weak social bonds is one-to-one gestures and vocalizations, as unlike grooming these behaviors do not require prolonged physical contact (Roberts et al., 2012b). However, one-to-one communication still requires some degree of close proximity and one-to-one prior visual attention (Roberts et al., 2014a) or brief tactile contact and thus a relatively low number of individuals can be bonded with at any one time. Moreover, these interactions are cognitively complex because animals have to remember the identities of the interactants and their past and present relationships with them to bond in an efficient manner. Thus, a signaling and bonding strategy of this type may not be effective in meeting the demands of maintaining social relationships in a large proximity network. In contrast, a larger-scale, vocally-based bonding system, such as a pant-hoot call, can be produced jointly by several individuals at the same time (Mitani and Nishida, 1993). In this context, simultaneous, rhythmically matched sound production and/or movement can replace the need for prolonged physical contact and act as an alternative bonding mechanism to grooming (Tarr et al., 2014). Here we therefore predict that the joint communication enables chimpanzees to bond effectively with the individuals beyond the size of the one-to-one grooming and communication network. Thus, there will be a switch from one-to-one grooming and communication to joint communication when the chimpanzees maintain large proximity networks. Such a communication system reduces the need for one-to-one interactions and therefore decreases the time and cognitive demands arising from one-to-one social bonding. How chimpanzees adjust their patterns of communication and grooming in proximity networks of differing sizes is thus informative of the key cognitive and time-budget pressures involved in sociality.

## Methods

### Study site and subjects

The Sonso community of East African chimpanzees (*Pan troglodytes schweinfurthii*) was observed at the Budongo Conservation Field Station, Budongo Forest Reserve in Uganda (latitude 1° 37′ −2° 00′N; longitude: 31° 22′ −31°46′E). Observations of communication and social relationships were conducted in September 2006, between April and July 2007 and March and June 2008. The data presented in this paper was collected during the rainy season between March and June 2008 (3.5 months), following subjects between 07:00 and 16:00 at least 5 days a week. All of the data were derived from the sample group of 12 focal subjects (6 adult males and 6 adult females). The key focus of this paper is the relationships between the variables of interest (proximity, grooming, and communication) from the random subsample of the larger community, rather than examining the properties of the social network as a whole. Thus whilst this data cannot be used to make inferences about the social network structure of the entire Sonso community, it can be used to examine predictors of proximity between pairs of chimpanzees and predictors of the size of proximity networks. Recent simulation analysis has shown that valid conclusions can be drawn about individual level social metrics based on a subset of the whole social network (Silk et al., 2015). Distance to the focal chimpanzee and limb injuries could influence the propensity to use gestural communication, and the type of gestural communication used. Therefore, we only selected focal chimpanzees for detailed behavioral observations that did not have any limb injuries and that were well habituated. We also selected focal chimpanzees so that all age and rank classes were equally represented in the sample—see Table 1 for demographic and sampling details of the focal chimpanzees. This sample was taken from the wider community which consisted of ~74 individuals: 21 adult females and 10 adult males. This research was approved by the University of Stirling Ethics Committee.

**Table 1:** **Demographic and sampling details of the study group**.

**Focal subject**	**Sex**	**Age (years)**	**Total observation duration (minutes)**
BB	Male	21	516
HW	Male	15	1030
KT	Male	15	1026
KU	Female	29	910
KW	Female	27	510
ML^a^	Female	33	1118
MS	Male	17	524
NB^a^, ^c^	Female	46	500
NK^b^	Male	26	582
RH	Female	43	1038
SQ	Male	17	554
ZM^a^	Female	40	710

a *oestrous female*;

b *alpha male*;

c *alpha female*.

### Data collection protocol

Quantitative focal animal follows were taken to establish a complete inventory of the patterns of social relationships and communication for each of the focal individuals. Chimpanzees travel each day, moving between different areas in their territory to access food. In this study we did not focus solely on chimpanzees occupying the same area but sampled chimpanzees from different areas, following them whenever they traveled. Focal subjects were chosen systematically and their behavior recorded during a standardized observation period. As far as possible, each focal chimpanzee was sampled equally at different times of the day and throughout the study period and we aimed to sample each focal individual at least once every week. In order to avoid dependency in the data set, we took consecutive samples of the same focal subject at least 20 min apart. We recorded the behavior of the focal chimpanzee and non-focal individuals who were present in the same party. The party was defined as the group of individuals within a spread of around 35 m. Behavioral data collected in this study came from five sources. First, we conducted 18 min focal follows which consisted of 9 scans at 2 min intervals of the activity of the focal individual and their association patterns (i.e., grooming given/received/mutual, identity of grooming partner, identity of individuals present within 10 m and more than 10 m away from the focal individual). Second, we continuously collected data on gestural communication to accompany the 18 min instantaneous sampling of associations and activity patterns in the chimpanzees. We recorded gestures continuously using a digital video camera recorder, with the camera centered on the focal animal but also taking a wider view to include interactants within the visible presence of the focal individual. For each instance of gestural behavior recorded, we described and recorded onto the camera the identity of the signaller and the recipient, the presence/absence of goal directedness, the response and the functional context of signal production. Additionally, we recorded the presence of any pant-grunt calls and the identity of the signaller and the recipient. Pant-grunts are a submissive vocalization in chimpanzees, so those individuals who receive more pant-grunt calls have a higher rank. Moreover, we noted pant-hoot calls accompanying the gestural communication. These calls can be produced and received by several group members simultaneously, therefore all of the individuals within 10 m of the signaller were identified as recipients of the pant-hoot calls. This data collection protocol allowed us to build up a detailed and accurate picture of the patterns of behavioral interactions (grooming, proximity, gestures, pant-hoot, and pant-grunt calls) in chimpanzees. The sampling of association patterns was conducted by an experienced field assistant, who was unaware of the aims of the study. The field assistants undergo an annual inter-observer reliability test, in order to maintain the consistency of scoring of the group composition and proximity across field assistants. The results of these tests are consistently above 0.85 Spearman's rank correlation coefficient, *r*_s_. The video recording of the gestures and calls was carried out by AR, whereas simultaneous collection of social context data was performed by the field assistant. Thus the data on association patterns and the gestural data were collected independently of each other and only considered together during the data processing and analysis.

### Video analyses of gestural communication

As a first step in the analysis, an inventory of gesture types was derived from the video recordings (Roberts et al., 2014a). For video analysis of gestures, footage was viewed on a television set and coded. We coded nonverbal behavior as an act of gestural communication if it was an expressive movement of the limbs or head and body posture that was mechanically ineffective, communicative (i.e., consistently produced change in the behavior of the recipient) and intentional. Following the criteria used in previous research (Hewes, 1973; Tomasello et al., 1984; Pika and Tomasello, 2002; Liebal et al., 2004; Leavens et al., 2005; Genty et al., 2009), we scored behavior as intentionally produced if (1) the signaller directed a gesture at a recipient and observed the recipient's response during and after the gesture, (2) the production of a gesture was sensitive to the recipient's visual attention state, (3) the signaller persisted in gesture production when the recipient failed to respond, (4) a gesture consistently elicited a change in recipient's behavior by non-mechanical means, (5) the gesture was produced in presence of the immediate audience. We evaluated these criteria for each gesture type and each intentionality criterion separately, using pooled data across subjects. If 60% of the cases of a particular nonverbal behavior type displayed at least one of the intentionality criterion listed, we considered that nonverbal behavior type to be an intentional gesture. We grouped gesture cases into gesture types qualitatively based on the objective judgment of similarity in morphology (i.e., presence/absence and type of head, trunk, arm movement; posture, social orientation). The description of repertoire with video clips for each gesture type can be found in Roberts et al. (2012b, 2014a). Gestures occurred in sequences, defined as one or more than one gesture made consecutively by one individual, toward the same recipient, the same goal, within the same context, within a maximum of 30 s interval. Moreover, to examine gestural communication in relation to social behavior, for each gesture event we recorded: the identity of the signaller (the individual performing a gesture); the identity of the recipient (individual at whom the gesture was most clearly directed, as determined from the orientation of head and body of the signaller during or immediately after performing a gesture, i.e., the signaller had the recipient within its field of view); the recipient's behavior after production of the gesture (response); the signaller's behavior prior to and after production of the gesture, and the accompanying context. On the basis of this information, gestures were grouped into functional categories. The second coder scored a random sample of 45 gesture sequences (10.42% of the total number of 432 gesture sequences) for the functional category of gesture, assigning them to one of the categories. The Cohen's Kappa coefficient showed that reliability was good for the gesture function (*K* = 0.70; Bakeman and Gottman, 1997).

### Behavioral measures

Tests of similarity in association patterns between scans and samples were conducted to ensure that the sampling protocol did not bias the results. Details of these analyses are provided in SI 1. The behavioral measures were then calculated in the following manner.

#### The dyadic association measure

The dyadic association measure (DA) is the duration of time focal subject A spent in close proximity (within 10 m) to non-focal subject B per hour spent in the same party, or:

$${\text{DA}}_{\text{AB}}=[{\left(\text{P}10_{\text{AB}}{}^{*}2\right)}^{*}60]/{\text{PSP}}_{\text{AB}}{}^{*}2$$

where P10_AB_ = the number of times A was in close proximity (within 10 m) to B

PSP_AB_ = the number of times A was in the same party as B

2 = duration of instantaneous subsample interval in minutes

60 = the number of minutes in an hour

Note that the multiplication by 60 enabled meaningful comparisons between indices (see below).

#### The dyadic association measure between kin

The dyadic association measure between kin (DAK) is the duration of time focal subject A spent in close proximity (within 10 m) to non-focal subject B, who is the maternal kin of A, per hour spent in the same party. No other kin relations were present among the focal subjects. The equation is:

$${\text{DAK}}_{\text{AB}}=[{\left(\text{P}10_{\text{AB}}{}^{*}2\right)}^{*}60]/{\text{PSP}}_{\text{AB}}{}^{*}2$$

where P10_AB_ = the number of times A was in close proximity (within 10 m) to B, who is related to A

PSP_AB_ = the number of times A was in the same party as B who is the kin

2 = duration of instantaneous subsample interval in minutes

60 = the number of minutes in an hour.

#### The dyadic association measure of the oestrous female

The dyadic association measure of the oestrous female (DAR) is the duration of time focal subject A who is a female exhibiting sexual swelling in the final phase of tumescence spent in close proximity (within 10 m) to non-focal subject B, per hour spent in the same party, or:

$${\text{DAR}}_{\text{AB}}=[{\left(\text{P}10_{\text{AB}}{}^{*}2\right)}^{*}60)]/{\text{PSP}}_{\text{AB}}{}^{*}2$$

where P10_AB_ = the number of times A (who is oestrous female) was in close proximity (within 10 m) to B

PSP_AB_ = the number of times A was in the same party as B

2 = duration of instantaneous subsample interval in minutes

60 = the number of minutes in an hour.

#### The dyadic grooming measure

The dyadic grooming measure (GA) is the duration of time focal subject A groomed with non-focal subject B when B was in close proximity (within 10 m) to focal subject A, per hour spent within 10 m of the non-focal subject B, or:

$${\text{GA}}_{\text{AB}}=[{\left({\text{GR}}_{\text{AB}}{}^{*}2\right)}^{*}60]/\text{P}10_{\text{AB}}{}^{*}2$$

where GR_AB_ = the number of times A groomed B when in close proximity (within 10 m) to B

P10_AB_ = the number of times A was in close proximity (within 10 m) to B

2 = duration of instantaneous subsample interval in minutes

60 = the number of minutes in an hour.

#### The dyadic communication measure

The dyadic communication measure (CA) is rate at which focal subject A communicated to non-focal subject B when B was in close proximity (within 10 m) to focal subject A, per hour spent within 10 m of the non-focal subject B, or:

$${\text{GA}}_{\text{AB}}=({\text{C}}_{\text{AB}}{}^{*}60)/\text{P}10_{\text{AB}}{}^{*}2$$

where C_AB_ = the number of times A communicated with B when in close proximity (within 10m) to B

P10_AB_ = the number of times A was in close proximity (within 10 m) to B

2 = duration of instantaneous subsample interval in minutes

60 = the number of minutes in an hour.

Because the dyadic association measure, and the grooming and communication measures, are based on different denominators, they are independent of each other. Thus the dyadic association measure reflects the tendency of pairs of chimpanzees to associate with each other in close proximity (within 10 m) when they have an opportunity to do so i.e., per hour spent in the same party. All the time pairs of chimpanzees spend within 10 m of each other is included in the denominator, regardless of whether they are grooming or not. In contrast, the grooming and communication measures reflect the duration or rate of these behaviors when pairs of chimpanzees are within 10 m of each other. Thus two pairs of chimpanzees may spend equal amounts of time within 10 m of each other, but pair A–B may spend a longer duration of time grooming than pair C–D, out of this total time spent within 10 m.

#### Attribute measures

To control for the influence of demography, factors such as age, kinship, sex, and reproductive state need to be taken into account when examining chimpanzees' propensity to associate with each other. We used genetic data from previous studies to classify pairs (dyads) of chimpanzees as kin or non-kin (Reynolds, 2005). In the wild, chimpanzees reach physical and social maturity between ages 15 and 16 years old (Goodall, 1986). The Sonso community is a long running study site and therefore the age of most adult chimpanzees in the community is known. We classified dyads of chimpanzees as belonging to the same (5 years or less age difference) or a different (above 5 years age difference) age class (Mitani et al., 2002). We also classified chimpanzee dyads according to reproductive similarity. The reproductive status of the female was scored on the basis of the female sexual swelling, which is the enlarged area of the perineal skin which varies in size over the course of the menstrual cycle. We recorded the reproductive status of the female as oestrous if during the observation period the female exhibited maximum tumescence and was observed mating with the males. All the focal males were observed to mate with females and were therefore all assumed to be reproductively active. We also classified the sex similarity of dyads of chimpanzees, based on observable morphological characteristics referring to sex. The full details of the categorization of attribute data are provided in SI Table 2.

### Social network analysis

Broadly, from the behavioral measures described above, different networks were created for each behavior. Each network matrix consisted of 12 rows and 12 columns, with each row and column denoting a different focal chimpanzee. The values in each cell of the matrix represented the value for that particular behavior for a specific pair of chimpanzees (e.g., the duration of grooming between Bwoba and Hawa, per hour spent in close proximity). These behavioral networks were weighted networks—that is each cell consisted of a continuous value representing that behavior, rather than a 1 or a 0 indicating the presence or absence of a tie. Further, the networks were directed in that the rate of gestures by Bwoba that were directed to Hawa may be different to the rate of gestures by Hawa that were directed to Bwoba. According to the type of analyses being carried out, these weighted, directed networks were sometimes transformed into binary or symmetrical networks, as described below.

From these network matrices, centrality measures were calculated, using normalized degree centrality (Croft et al., 2010). Normalized degree centrality is the average value of each row or column of the network matrix i.e., the average value of that behavior for each focal chimpanzee. Because the network is directed, in degree and out degree were calculated separately. Out degree refers to behaviors directed by the focal chimpanzee to conspecifics, whilst in degree refers to behaviors directed by conspecifics toward the focal chimpanzee. We used degree to measure centrality rather than eigenvector centrality or beta centrality as these latter two measures incorporate the effects of indirect links on a focal node. Thus for eigenvector and beta centrality, the centrality of chimpanzee A depends not just on the direct ties chimpanzee A has with conspecifics B, C, and D, but also the ties chimpanzees B, C, and D have with others. For the purposes of this analysis, degree centrality provides a clearer indication of the direct connectedness of focal chimpanzees to conspecifics in the network and the likely costs of maintaining these relationships, rather than also taking into account indirect network connections. Further, recent simulation analysis demonstrated that when only part of a network is sampled, simple measures of centrality such as degree are more reliable than more complex measures of centrality such as betweenness or eigenvector centrality, which are more dependent on accurately measuring network structure (Silk et al., 2015). This is especially the case when the network is relatively small, as the Sonso community of chimpanzees is. Thus, whilst in future work with a more complete network of a whole chimpanzee community it would be interesting to examine how communication relates to these more complex measures of centrality, for these analyses degree centrality was used.

All data transformations and analyses were carried out using UCINET 6 for Windows (Borgatti et al., 2014). For the comparison of normalized mean degree across the four main behavioral networks (proximity, gesture, grooming, and pant-grunts), we dichotomized and symmetrized the networks (Borgatti et al., 2013). This allows for easier interpretation of the normalized mean degrees, which refer to the mean proportion of all possible ties which are present. For dichotomization, all values over zero were scored as 1 (present) and all values of zero were classed as absent. For symmetrization, a tie was scored as present if there was a 1 in either of the two cells corresponding to each pair of individuals (cell *i, j* or cell *j, i*).

The observations that make up network data are not independent of each other and thus in general standard inferential statistics cannot be used on network data. Instead, a set of analyses using randomization (or permutation) tests have been developed where the observed value is compared against a distribution of values generated by a large number of random permutations of the data. The proportion of random permutations in which a value as large (or as small) as the one observed is then calculated, and this provides the *p*-value of the test (Borgatti et al., 2013). We used Multiple Regression Quadratic Assignment Procedure (MRQAP) to examine the relationships between the different behavioral networks (Borgatti et al., 2013). MRQAP regression is similar to standard regression in that it allows for the examination of the effect of a number of predictor variables (e.g., grooming network, gestural communication network) on an outcome variable (proximity network). Several different types of MRQAP regression are available and we used Double Dekker Semi-Partialling MR QAP regression, which is robust against the effects of network autocorrelation and skewness in the data (Dekker et al., 2007). The number of permutations used in this analysis was 2000.

Whereas MRQAP regression is used to examine the association between different networks, node-level regressions are used to examine the predictors of individual differences. In our analyses, these individual differences related to proximity—we examined which behaviors are associated with individual chimpanzees having a larger number of strong proximity bonds. Thus we assessed the effect of a number of predictor variables (e.g., the out degree for gestures, sex of focal chimpanzee) on a single outcome variable (proximity in degree) using 10,000 random permutations. For these analyses, both reciprocated and non-reciprocated strong proximity bonds were considered (dyads of individuals who had values of proximity association equal or greater than the mean plus half SD were scored as “strong ties”) to account for the number of the individuals in close proximity.

Finally, we used Geary's C statistic to assess the autocorrelation between attribute data (e.g., the total duration of observation) and network data (e.g., proximity network). When there is no association between variables, the Geary statistic has a value of 1.0, with values of <1.0 indicating a positive association and values over 1.0 indicating negative association.

## Results

### Can grooming and communication predict proximity networks?

In this study we examined a mean of 12.52 (range 8.33–18.63) h of independent focal data across 12 individual subjects (Table 1). This is in accordance with the sample size obtained in other studies of primate gestural communication and social dynamics (Pollick and de Waal, 2007). There was no statistically significant relationship between the duration of observation for dyads and rates of proximity or communication, suggesting a sufficient sampling duration. The details of this analysis are provided in SI 3.

Across the 132 dyads, each chimpanzee dyad spent a mean of 21.16 (range 0–60) min in close proximity (within 10 m) with conspecifics, per hour spent in the same party. In the overall close proximity network, the chimpanzees were connected to almost all other focal individuals—95.5% of potential connections to group members were present (range 82–100%). Thus there was at least some level of proximity between almost all chimpanzee dyads. In terms of the behavioral measures, per hour spent in close proximity, chimpanzees produced a mean of 2.20 (range 0–60) gesture sequences directed at the partner and groomed with the partner for 1.73 (range 0–30) min. The mean degree of the grooming network (the percentage of potential connections chimpanzees had with others) was 36.4% (range 9–91%) and the mean degree was 56% (range 18–100%) for the gesture network. The ethogram for the gesture functions and their accompanying gesture types, as well as the definitions of grooming sub-categories (grooming given, received, and mutual), are provided in Table 2. The details of the rate of production and mean degrees of grooming mutual, received, given, and gestures per function are given in Table 3.

**Table 2:** **Ethogram for gesture functions, grooming categories and accompanying gesture types**.

**Gesture function/grooming category**	**Description**	Gesture types^*^
Threat to dominate	Individual performs intimidating gestures toward the recipient, where there is no clear reason for the conflict of interest, but the recipient reacts by being frightened (e.g., responds by uttering screams or a pant-grunt vocalizations).	Dangle, Stationary stiff, Stamp quadrupedal, Walk stiff, Swagger quadrupedal, Swagger bipedal, Jump, Run stiff, Swing, Unilateral swing, Stiff extend, Shake stationary, Shake mobile, Break, Arm flap
Food sharing	Gestures directed by the signaller at the recipient in anticipation of sharing valuable food, when the food is possession of the recipient and in clear view of the signaller.	Vertical extend
Other threat	Individual performs aggressive or rejection gestures, where there is a clear conflict of interest over a resource or behavior. These include refusals to reassure another, threats to displace another from resource such as food, threats to punish for aggression toward third party, threats to retaliate against aggression toward self or activity toward third party (e.g., copulation); threats to redirect aggression received from someone else.	Lunge, Bob, Stationary stiff, Turn head, Tip head, Stamp quadrupedal, Drum, Walk stiff, Swing, Swagger bipedal, Run stiff, Jump, Crouch walk, Crouch run, Vertical extend, Tap object, Shake stationary, Shake mobile, Knock, Forceful extend, Arm flap, Stroke short
Travel	Gestures performed to induce recipient to follow signaller by walking or running to depart from current location toward another.	Dangle, Bounce, Stationary stiff, Stamp sitting, Stamp quadrupedal, Drum, Walk stiff, Swagger bipedal, Run stiff, Jump, Swing, Unilateral swing, Stiff extend, Shake stationary, Shake mobile, Beat
Copulation	Gestures accompanied by penile erection, directed toward a fully tumescent female, which elicit approach for mating.	Bounce, Turn back, Stationary stiff, Present rump, Present mount, Present genitals, Hold object, Clip by mouth, Stamp sitting, Stamp quadrupedal, Walk stiff, Crouch walk, Jump, Vertical extend, Touch self, Shake stationary, Shake mobile, Linear sweep, Hit object, Clip by hand, Arm raise, Arm beckon, Wipe, Unilateral swing
Reassurance	Individual gestures toward the recipient, who seems distressed, frightened or hurt by signallers own behavior or third party threat.	Stationary stiff, Stand tandem, Present rump, Run stiff, Locomote tandem, Crouch run, Vertical extend, Touch long, Touch backhand, Offer hand, Embrace, Rub
Greeting	Individual gestures when approaching, being approached or leaving approach with the recipient, when recipient is non-antagonistic or when the recipient or third party distressed, frightened or hurt the signaller.	Thrust genitals, Slide, Rock, Push by rump, Lunge, Drag self, Dangle, Bob, Turn back, Present torso, Present rump, Crouch, Bow, Stand tandem, Stroke by mouth, Sniff, Smack lip, Nod, Kiss, Bite, Swing, Run stiff, Locomote tandem, Jump, Crouch walk, Crouch run, Vertical extend, Touch long, Touch backhand, Tap another, Stretched extend, Stiff extend, Linear sweep, Limp extend, Hand bend, Grab, Embrace, Pull another, Hold hands
Gesture to mutually groom	Invitation for groom (using one or both hands individual pushes another's hair back with the thumb or index finger to pick at the exposed skin, removing parasites), which results in both individuals grooming each other at the same time.	Present torso, Smack lip, Limp extend, Arm raise
Gesture to receive groom	Invitation for groom, which results in signaller receiving grooming from the recipient.	Roll over, Present torso, Smack lip, Lower head, Present leg, Touch backhand
Gesture to give groom	Invitation for groom, which results in signaller grooming the recipient.	Present torso, Smack lip, Touch backhand, Push by hand, Pull another, Limp extend, Touch innerhand
Play	Individual performs gesture toward the recipient, to induce another to wrestle, chase or tickle in non-agonistic relaxed manner.	Tickle, Shake limb, Rub, Offer hand
Synchronized low-intensity pant-hoot	Pant-hoot call produced jointly with other group members and accompanied by simultaneous production of visual gestures, which can be perceived only by looking at signaller.	Dangle, Run stiff, Crouch walk, Arm flap
Solo high-intensity pant-hoot	Pant-hoot call produced solo (without joining in by other group members) and accompanied by simultaneous production of auditory gestures, which produce sounds audible at a distance of at least 10 meters independently of the acoustic properties of the pant-hoot call. If both visual and auditory gestures simultaneously accompanied the pant-hoot call it was scored as high-intensity.	Bounce, Dangle, Sway, Drum, Stamp quadrupedal, Run stiff, Swagger stationary, Swing, Walk stiff, Pound, Shake mobile, Shake stationary, Linear sweep, Slap self
Synchronized high-intensity pant-hoot	Pant-hoot call produced jointly with other group members and accompanied by simultaneous production of auditory gestures, which produce sounds audible at a distance of at least 10 meters independently of the acoustic properties of the pant-hoot call. If both visual and auditory gestures simultaneously accompanied the pant-hoot call it was scored as high-intensity.	Rock, Stationary stiff, Drum, Stamp quadrupedal, Crouch walk, Run stiff, Swagger bipedal, Swagger quadrupedal, Swing, Walk stiff, Beat, Pound, Shake mobile, Shake stationary
Grooming mutual	Focal individual simultaneously grooms with non-focal subject	
Grooming received	Focal individual receives grooming from non-focal subject	
Grooming given	Focal individual grooms non-focal subject	

* *Description and video footage of gesture types can be found in Roberts et al. (2012b, 2014a)*.

**Table 3:** **Rate of production and mean degree of grooming and communication networks between *N* = 12, 132 chimpanzee dyads**.

**Behavior**	**Mean duration (grooming in minutes) or frequency (sequences of communication) per hour spent in close proximity**	**Overall range (frequency/duration of communication/grooming per hour spent in close proximity)**	**Mean degree (% of potential connections with others)**	**Overall range mean degree (%)**
**GROOMING**				
Grooming given	0.69	0–18.75	27.2%	0–64
Grooming received	0.53	0–15.56	21.2%	0–73
Grooming mutual	0.66	0–20	16.6%	0–55
**COMMUNICATION**				
Threat to dominate	0.07	0–7.50	6%	0–27
Food sharing	0.002	0–0.36	1.5%	0–9
Other threat	0.07	0–3.75	13.6%	0–36
Travel	0.034	0–3.75	3%	0–9
Copulation	0.14	0–8.05	10.6%	0–27
Reassurance	0.08	0–10	3%	0–18
Greeting	0.27	0–3.91	30.6%	9–100
Gesture to mutually groom	0.07	0–7.50	9.1%	0–36
Gesture to receive groom	0.20	0–7.50	19.7%	0–55
Gesture to give groom	0.37	0–17.50	15.1%	0–46
Play	0.17	0–22.94	1.5%	0–9
Synchronized low-intensity pant-hoot	0.049	0–4	10.6%	0–27
Solo high-intensity pant-hoot	0.08	0–5	12.1%	0–36
Synchronized high-intensity pant-hoot	0.20	0–10	18.2%	0–36
Pant-grunt	0.33	0–5.45	33.3%	9–91

We used MRQAP to examine how rates of gestural communication for chimpanzee dyads predicted the duration of time the pair of chimpanzees spent in close proximity, per hour in spent in the same party. In all of these analyses we controlled for differences in age, sex, kinship, and the reproductive state between dyads. Details of all models, including insignificant findings, are provided in SI Tables 4–15. Overall, chimpanzee dyads that spent longer in close proximity had higher rates of all types of gesture sequences combined (*r*^2^ = 0.055, β = 0.183, *p* = 0.026). We then examined how rates of sequences of gestures, categorized according to the function, predicted the duration of time spent in close proximity. First, we examined whether rates of threat to dominate gestures predicted duration of time spent in close proximity, independently of other behaviors. Chimpanzees who were more likely to spend time in close proximity had a significantly higher rate of sequences of threat to dominate gestures (*r*^2^ = 0.049, β = 0.162, *p* = 0.029) than the chimpanzees who associated with each other less frequently.

We next used a MRQAP regression model to examine the predictors of duration of time spent in close proximity taking into account all gestures and grooming in one model (Table 2). Chimpanzees who are more likely to spend time in close proximity used greetings (*r*^2^ = 0.242, β = 0.173, *p* = 0.018), gestures relating to mutual grooming (*r*^2^ = 0.242, β = 0.908, *p* = 0.048), and low-intensity pant-hoots (*r*^2^ = 0. 242, β = 0.175, *p* = 0.017) at a higher rate, and also received grooming at a higher rate (*r*^2^ = 0. 242, β = 0.215, *p* = 0.017). Synchronized high-intensity pant-hoots (*r*^2^ = 0. 242, β = −0.171, *p* = 0.015) and reassurance gestures (*r*^2^ = 0.242, β = −1.040, *p* = 0.032) were significantly negatively correlated with duration of time spent in close proximity.

The weighted network proximity matrices cannot distinguish between “reciprocated” and “one-sided” relationships and therefore we classified proximity between chimpanzee dyads in a binary way, based on established methods other researchers have used to identify different social partners in primates (Gilby and Wrangham, 2008; Kanngiesser et al., 2011). First, chimpanzee dyads who had values of proximity association equal or above the mean plus half SD (i.e., who spent 30.3 or more minutes in close proximity, per hour spent in the same party), were scored as 1 if the proximity was reciprocated (i.e., both A to B and B to A had values of close proximity equal to or above 30.3 min duration). These bonds we termed “preferred reciprocated close proximity bonds.” Dyads where one or both parties had a proximity duration of below 30.3 min duration were scored as 0. Chimpanzees had preferred reciprocated close proximity bonds with only a small number of the individuals. In the binary network based on preferred reciprocated close proximity bonds, only 15.1% of potential connections were present (range 0–46%). Second, dyads of individuals who had values of proximity association equal or greater than the mean plus half SD, were scored as 1 when the proximity was non-reciprocated (i.e., only A to B but not B to A had duration of proximity association equal or above the 30.3 min—“preferred, non-reciprocated close proximity bonds”), whereas other dyads were scored as 0. In this network 37.9% of all potential close proximity connections were preferred but not reciprocated (range 18–55%). Third, dyads of individuals who had values of proximity association equal or below the mean minus half SD (who spent 16.23 or less minutes in close proximity to each other per hour spent in same party), were scored as 1 (“non-preferred close proximity bonds”), whereas other dyads were scored as 0. Chimpanzees had non-preferred close proximity bonds with 53.01% of potential proximity connections (range 9–82%).

**Figure 1:**
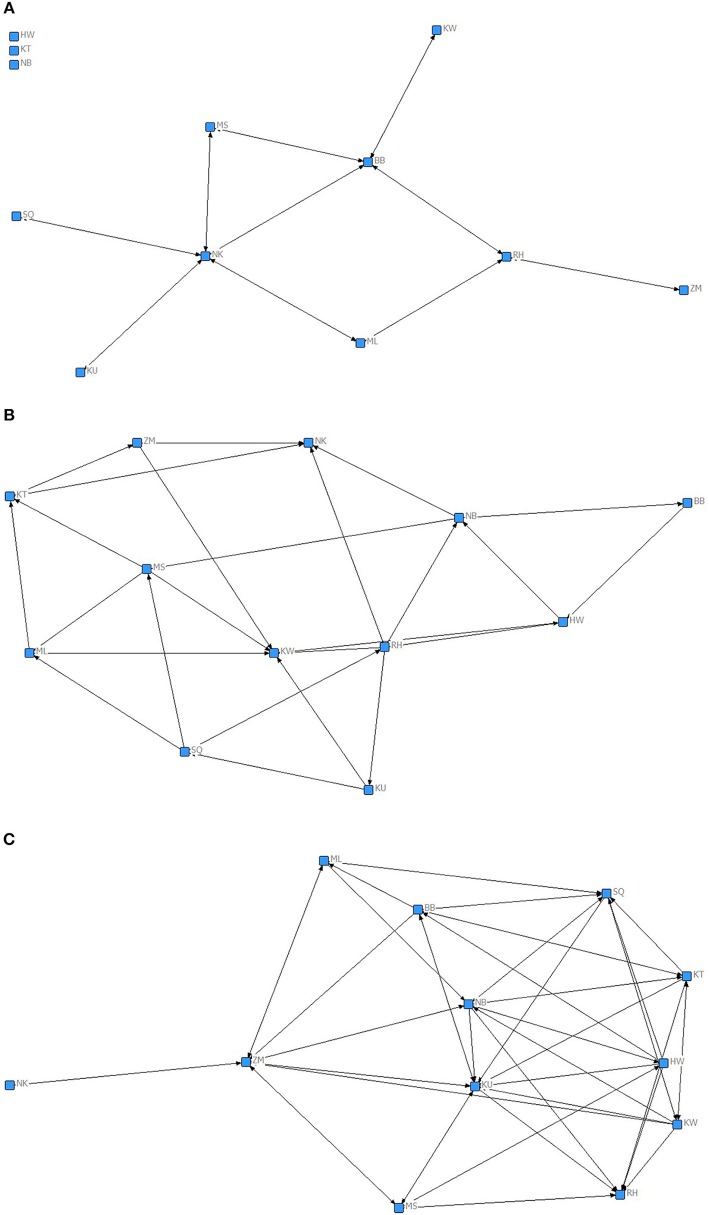
**Chimpanzee proximity network based on (A)** preferred, reciprocated close proximity bond, where A to B and B to A dyads had values of close proximity equal or above mean plus half SD (30.3 min duration per hour spent in same party); **(B)** preferred, non-reciprocated close proximity bond, where A to B but not B to A dyads had values of close proximity equal or above mean plus half SD (30.3 min duration per hour spent in same party); **(C)** non-preferred close proximity bond, where A to B dyads had values of close proximity equal or below the mean minus half SD (16.23 min duration per hour spent in same party). Nodes represent individual chimpanzees. Lines indicate the presence of a given bond between a particular dyad (arrow heads indicate the direction).

**Figure 2:**
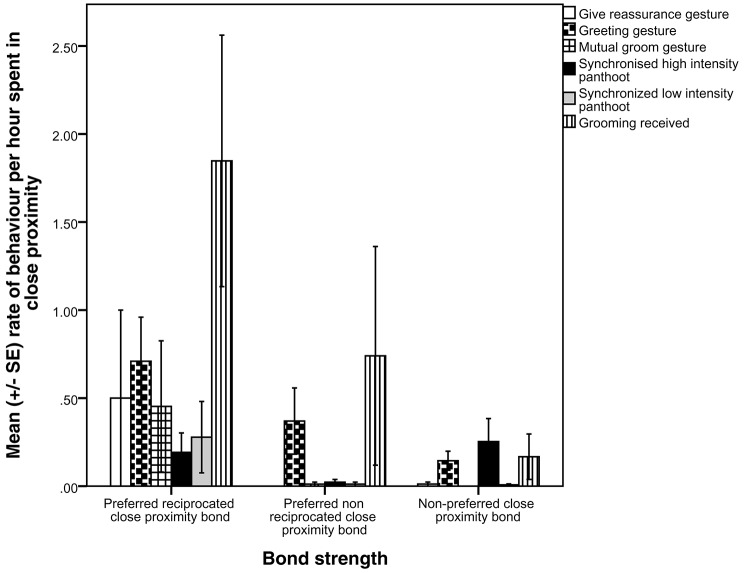
**Mean rate of gestures across function and grooming categories, per close proximity bond strength**.

We used MRQAP regression models to examine the predictors of the presence of proximity bonds, including all gestures and grooming in one model (Table 2). Visualizations of the binary proximity networks are provided in Figures 1A–C and the mean rate of gestures across function and grooming categories by close proximity bond strength are shown in Figure 2. Chimpanzees more likely to have a preferred reciprocated close proximity bond used greetings (*r*^2^ = 0.471, β = 0.162, *p* = 0.034), gestures relating to mutual grooming (*r*^2^ = 0. 471, β = 1.579, *p* = 0.001), gestures related to receiving grooming (*r*^2^ = 0. 471, β = 0.707, *p* = 0.001), travel (*r*^2^ = 0. 471, β = 0.226, *p* = 0.005), and synchronized low-intensity pant-hoots (*r*^2^ = 0. 471, β = 0.258, *p* = 0.001) at a higher rate. Threat to dominate gestures (*r*^2^ = 0. 471, β = −0.492, *p* = 0.027), reassurance gestures (*r*^2^ = 0. 471, β = −1.466, *p* = 0.001), and gestures to play (*r*^2^ = 0. 471, β = −0.104, *p* = 0.046) negatively predicted the presence of a preferred reciprocated close proximity bond. Gestures to receive groom (*r*^2^ = 0. 107, β = −0.463, *p* = 0.012) and synchronized high-intensity pant-hoot (*r*^2^ = 0. 107, β = −0.114, *p* = 0.024) negatively predicted presence of preferred, non-reciprocated close proximity bond. Finally, chimpanzees more likely to have non-preferred close proximity bond used synchronized high-intensity pant-hoot (*r*^2^ = 0. 229, β = 0.189, *p* = 0.006) at a higher rate. Other threat (*r*^2^ = 0. 229, β = −0.146, *p* = 0.010), copulation (*r*^2^ = 0. 229, β = −0.154, *p* = 0.007), greetings (*r*^2^ = 0. 229, β = −0.220, *p* = 0.002), synchronized low intensity pant-hoot (*r*^2^ = 0. 229, β = −0.126, *p* = 0.020), and grooming received (*r*^2^ = 0. 229, β = −0.204, *p* = 0.002) were negatively associated with non-preferred close proximity bond.

### Pant-grunts and proximity network

We examined the relationships between pant-grunt vocalizations, gestural communication, and proximity. Chimpanzees more likely to spend time in close proximity used pant-grunts at a higher rate (*r*^2^ = 0.066, β = 0.209, *p* = 0.010). When examining rates of gestural communication according to function and grooming as predictors of pant-grunt given, greeting gestures were positively correlated with pant-grunt vocalizations (*r*^2^ = 0.807, β = 0.209, *p* = 0.001) whereas reassurance gestures (*r*^2^ = 0.807, β = −0.363, *p* = 0.049) were negatively correlated.

### Predictors of proximity centrality

We created a binary proximity network, where dyads of individuals who had values of proximity association equal or above the mean plus half SD, were scored as 1 (“preferred close proximity partners”), whereas the remaining dyads were scored as 0. In the network of preferred close proximity bonds, only 34.1% of potential connections were present (range 18–82%). We calculated the normalized degree centrality for each individual chimpanzee, i.e., the average value of each row or column of the preferred close proximity bonds matrix. When networks are directed, in degree and out degree are calculated separately. Out degree refers to behaviors directed by the focal chimpanzee to conspecifics, whilst in degree refers to behaviors directed by conspecifics toward the focal chimpanzee. This proximity network was directed because some bonds were not reciprocated in this analysis and therefore in degree was calculated and used in all models. For communication and grooming networks normalized degree (the proportion of all potential connections chimpanzees had with others) was used.

**Figure 3:**
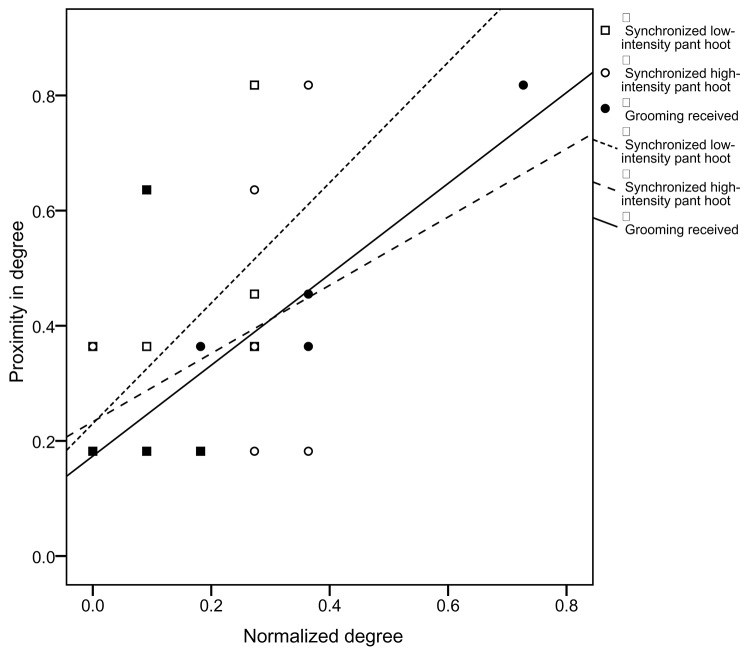
**Relationship between proximity in degree and normalized degree (proportion of potential connections present) for pant-hoots and grooming across 12 focal chimpanzees**. Grooming received shown with filled circles and solid line, synchronized low-intensity pant-hoot shown with open circles and dotted line and synchronized high-intensity pant-hoot shown with squares and dashed line.

The analyses used node-level regressions to examine the predictors of proximity in degree. All of these analyses controlled for the duration of time spent in proximity to oestrus females, time spent in proximity to kin, and the age and sex of the focal chimpanzee. Overall chimpanzees with a high proximity in degree had a high degree of gesture sequences combined (*r*^2^ = 0.422, β = 0.697, *p* = 0.033) and a high degree of pant-grunt given (*r*^2^ = 0.463, β = 0.688, *p* = 0.028). We examined the relative roles of gestures identified by previous models as positively (grooming received, greetings, gestures to mutually groom, synchronized low-intensity pant-hoot) or negatively (synchronized high-intensity pant-hoot, reassurance) associated with duration of time spent in close proximity in predicting proximity in degree (Figure 3). The only positive predictor of proximity in degree was the rate of synchronized high-intensity pant-hoots (*r*^2^ = 0.908, β = 2.892, *p* = 0.024) and grooming received (*r*^2^ = 0.908, β = 2.830, *p* = 0.047), with greetings negatively associated with proximity in degree (*r*^2^ = 0.908, β = −2.695, *p* = 0.029). Finally, we examined the relative roles of gestures identified by previous models as positively (greetings, gestures to mutual groom, receive groom, travel, low-intensity pant-hoot) or negatively (threat to dominate, reassurance, and gestures to play) associated with preferred, reciprocated close proximity bonds in predicting proximity in degree (Figure 3). The only positive predictor of proximity in degree was the degree of synchronized low-intensity pant-hoots (*r*^2^ = 1, β = 4.994, *p* = 0.031), with all other categories of gestures not statistically significant. All *p*-values reported in this study are uncorrected for multiple comparisons and would therefore not survive a conservative Bonferroni correction.

## Discussion

It has long been established that primates use grooming as a mechanism to maintain their social relationships, but the role of communication in maintaining social relationships is less well understood. This study used social network analysis to examine how grooming, gestures and vocalizations were associated with social bonds (as measured by time spent in close proximity per hour spent in the same party) in wild chimpanzees. There were three key findings.

First, we examined the extent to which chimpanzees preferentially associated and interacted with specific individuals, in terms of time spent in close proximity, rates of grooming and rates of communication. Chimpanzees were connected through close proximity to some extent with almost all (over 95%) of the group members but maintained high levels of close proximity to a much smaller number of group members. Further chimpanzees directed gestural communication to just over half (56%) of conspecifics and groomed just over a third (36%) of conspecifics. Thus chimpanzees did not interact with all individuals they found themselves in close proximity (within 10 m) to, but instead gestured and groomed with specific individuals. This demonstrates that chimpanzees had distinct patterns of interaction with different members of their group, suggesting a differentiated set of social relationships. Whilst it is well established that primates preferentially groom with preferred social partners (Crockford et al., 2008; Lehmann and Boesch, 2008; Mitani, 2009; Foerster et al., 2015) this is the first study to demonstrate that vocal and gestural communication is also preferentially directed at specific social partners.

Second, we examined the predictors of close proximity between pairs of chimpanzees i.e., what predicts the duration of time Chimpanzee A spends in close proximity (within 10 m) to Chimpanzee B per hour spent in the same party? As expected, chimpanzees that had high levels of close proximity also had higher rates of grooming. Chimpanzee dyads that had high levels of close proximity also had a higher rate of dominance aggression gestures, indicating a cost to sociality (Dunbar, 2012). However, a specific set of affiliative signals—greetings, gestures to mutually groom and synchronized low-intensity pant-hoots—also predicted proximity between pairs of chimpanzees. The co-occurrence of these signals and grooming suggests that they may function as a form of “grooming at a distance,” exploiting on a larger scale the psychopharmacological mechanisms as those involved in grooming behavior (Dunbar, 1996; Tarr et al., 2016). When these signals were included in the model, dominance aggression gestures were no longer a significant predictor of proximity. Further, the pant-grunt call accompanying greeting gestures, which was previously suggested to reduce the risk of aggression (Goodall, 1986), also predicted proximity between pairs of chimpanzees. Conversely, communication related to dominance such as reassurance gestures (Faraut et al., 2015) and synchronized high intensity pant-hoots were negatively correlated with close proximity. These results demonstrate that in addition to grooming, affiliative communication appears to play a key role in relation to the maintenance of proximity and may be associated with reduction in the levels of aggressive communication between pairs of chimpanzees. Thus, chimpanzees appear to use a differentiated communication system, including both gestures and vocalizations, to maintain a differentiated set of social relationships. Affiliative communication and grooming is associated with preferred close proximity ties, whereas dominance communication is associated with non-preferred close proximity ties between pairs of chimpanzees.

The third key finding related to predictors of individual variation in proximity in degree, i.e., the extent to which other chimpanzees were found in close proximity to the focal chimpanzee. A high proximity in degree was associated with a higher rate of pant-grunt vocalizations, which are an indicator of high rank (Goodall, 1986). When the specific communication and grooming behaviors that were identified in previous models as related to overall proximity were examined in relation to proximity in degree, only the rate of grooming received and the rate of synchronized high-intensity pant-hoots predicted proximity centrality. This suggests that when individual chimpanzees are found in close proximity with numerous conspecifics, in addition to grooming, synchronized vocalizations accompanying loud auditory gestures may play a key role in managing these more numerous social relationships.

However, the weighted network matrices cannot adequately describe the differences between “reciprocated” and “one-sided” close proximity bonds. We therefore examined the predictors of reciprocated close proximity between pairs of chimpanzees i.e., what predicts the presence of a reciprocated close proximity bond between Chimpanzee A and Chimpanzee B? In previous research, we have shown that the presence of a reciprocated close proximity bond is predicted by a longer duration of mutual grooming and received grooming between pairs of chimpanzees (Roberts and Roberts, 2016). Here we extended these findings and showed that a specific set of signals—greetings, gestures to initiate mutual grooming, gestures to initiate receiving grooming, gestures to initiate travel, and synchronized low intensity pant-hoot—predicted the presence of a reciprocated close proximity bond between pairs of chimpanzees. When these signals were included in the model, grooming was no longer a significant predictor of close proximity. These results suggest that the time and cognitive constraints on grooming behavior may impose greater limits on social bonding than the constraints imposed by gestural communication. We also examined the extent to which the specific set of signals that predicted the presence of a reciprocated close proximity bond was related to proximity in degree. In this analysis, only synchronized low intensity pant-hoots accompanying visual gestures were significantly related to proximity in degree. Thus, for individual chimpanzees found in close proximity to numerous conspecifics with whom they had reciprocated bonds, synchronized vocalizations accompanying visual gestures appear to play particularly important role in communicating with these social partners.

The strategies described above may enable chimpanzees to manage a larger set of social relationships more effectively than would be possible through grooming alone. Previous studies showed that grooming plays a key role in regulating proximity in primates (Dunbar, 1991, 2010) and grooming has been used as a key indicator of social bonds in chimpanzees (Lehmann and Boesch, 2008; Mitani, 2009; Foerster et al., 2015). In this chimpanzee community, grooming co-varied with close proximity and chimpanzees who were central in the network received grooming from others for a longer duration. However, primates are limited in the amount of time they can devote to grooming, and typically focus a large proportion of their grooming on a small number of individuals (Dunbar, 1991; Kudo and Dunbar, 2001; Lehmann and Boesch, 2008; Mitani, 2009; Foerster et al., 2015). Thus, in larger social groups such as those of chimpanzees, relying on grooming alone to maintain social relationships may not be enough to maintain group cohesion (Dunbar, 1993; McComb and Semple, 2005). For the first time, our results show chimpanzees with higher levels of proximity not only have higher rates of grooming, but also have higher rates of gestural communication, per hour spent in close proximity. The advantage of gestural communication over grooming is that it can take place when pairs of chimpanzees are spatially separated, and thus may act as a more time efficient bonding mechanism than grooming. Further, a specific set of gestures were associated with close proximity, namely greetings and gestures used to initiate mutual grooming. These gestures may serve to reduce the levels of aggression and mitigate its effects when chimpanzees are forced into close proximity due to the clumped nature of the food resource (Wrangham, 1980; White and Wrangham, 1988) and efficiently indicate to conspecifics the affiliative intentions of the focal individual. Once this proximity has been established and regulated by gestural communication, grooming may be used to reinforce the social bond, but by its nature grooming cannot take place before proximity has been established.

However, gestural communication still relies on one-to-one communication, and is only effective over a short distance and thus only small number of individuals can be bonded in this manner. Here we show that vocally-based joint affiliation in the form of synchronized, low-intensity pant-hoots may help to overcome this constraint, influencing the proximity of individuals beyond the network of direct interactants. The potential bonding function of the pant-hoot call has also been shown in a recent study on wild chimpanzees, where on the day of signaling, pant-hoot chorusing predicted affiliative behaviors such as reciprocated grooming and coalitions (Fedurek et al., 2013). In contrast, aggressive coalitions and joint nonvocal displays where not higher on the days that the dyad was involved in reciprocated grooming, suggesting that pant-hoot chorusing may be a more effective indicator of short-term affiliations than grooming (Fedurek et al., 2013). Synchronized low-intensity pant-hoots are an even more effective way than grooming or gestural communication of affiliating with a larger number of chimpanzees simultaneously and coordinating group movements and thus may be effective at maintaining social cohesion. Synchronized low intensity pant-hoots may have similar psychopharmacological underpinnings as those underlying grooming behavior (Tarr et al., 2015; Weinstein et al., 2016) and could therefore be effective at bonding with a larger number of bonded individuals over longer periods (Dezecache and Dunbar, 2012).

Although a higher rate of synchronized low intensity pant-hoots are associated with the presence of reciprocated close proximity bonds, other types of vocalizations may be better suited to managing a larger set of weaker, non-reciprocated social bonds. Here we show that joint coalitionary aggression in the form of synchronized high intensity pant-hoots may be one way in which these relationships are maintained. On the behavioral level, aggressive signals, particularly those that are deep, sharp, sudden, and high volume gestures, are associated with high arousal and induce arousing, fear reactions in the recipients (Bryant, 2013; Roberts and Roberts, 2015, 2016). On the physiological level, aggressive signaling can affect the recipient's nervous system by inducing an increase in plasma cortisol release (Beerda et al., 1998). However, by joining in the aggression itself through the bouts of synchronized high intensity pant-hoots, recipients can reduce the negative impact of aggression on their nervous system (Arrowood, 1988). Such synchronized aggression may induce a convergence of joint emotional state with the signalers (Dezecache et al., 2015) thus reducing the stress arising from close proximity and also reducing the risk of being a recipient of aggression. Visual gestures accompanying low-intensity pant-hoot interactions require dyadic adjustment and possibly mutual visual contact to achieve synchrony and social bonding. In contrast, loud auditory gestures such as drumming can provide rhythmic scaffolding for the individuals to achieve synchrony through pant-hoots in large close proximity networks (Tarr et al., 2014). Such a system may reduce the cognitive load of monitoring and remembering of specific weak-tie identities and relationships by reducing the need for dyadic, one-to-one interaction. Aggressive communication can also introduce order in social relationships by firming up rank relationships and reducing the need to reinforce order through aggression (Flack et al., 2006; Beisner and McCowan, 2013). However, since high-intensity pant-hoots are arousing they may only be effective in maintaining relationships over short time periods. Overall, a complex communication system, comprising of a large and varied repertoire of gestures and vocalizations, appears to allow chimpanzees to manage a differentiated set of social relationships more effectively than by using grooming alone. One limitation of these findings is that we did not take multiple comparisons into account and only reported uncorrected results. However, the current study examined and provides support for specific hypothesis formulated a priori.

The finding that communication and proximity are interrelated may shed new light on the mechanisms of social bonding in our hominin ancestors. As time constraints limit the amount of time available for grooming, it has been theorized that as group size increased through human evolution, affiliative vocalizations, and then language played a central role in maintaining social bonds and group cohesion (Aiello and Dunbar, 1993). Our results suggest that in addition to grooming, gestures and synchronized vocalizations may have played key roles in maintaining social cohesion and reducing the aggression that can arise from close proximity. An increasingly complex and varied communication network may have enabled larger groups of hominins to maintain social cohesion and coordinate their activities, thus acting as an alternative bonding mechanism to grooming. Our results therefore suggest that a key function of both gestures and vocalizations in hominin evolution may have been social bonding and maintaining social cohesion in large social groups.

## Author contributions

SR, AR, designed the study, wrote the manuscript, and analyzed the data. AR designed the methods, collected the data, and coded the footage.

## Funding

This study was funded by the Economic and Social Research Council and a University of Stirling Fellowship to AR.

### Conflict of interest statement

The authors declare that the research was conducted in the absence of any commercial or financial relationships that could be construed as a potential conflict of interest.
