# Supplementary material for: Social Brain Hypothesis: Vocal and Gesture Networks of Wild Chimpanzees
Source: Front Psychol. 2016 Nov 24;7:1756. doi: 10.3389/fpsyg.2016.01756 (PMC5121241; doi:10.3389/fpsyg.2016.01756)
Supplement: Supplementary file 1 [file Table1.DOCX]

SUPPLEMENTARY INFORMATION

**Social brain hypothesis, vocal and gesture networks of wild chimpanzees**

**Authors:** Anna Ilona Roberts, Sam George Bradley Roberts

**SI 1 Sampling dependence**

In order to reduce pseudoreplication, we aimed to sample each focal subject when party had a unique composition, i.e. there was a change in composition of either focal males or females from the time the last subsample of preceding focal follow was taken. The proximity and grooming scans were taken 2 minutes apart during 18 minute sample duration. The samples of each consecutive focal subject were taken at least 20 minutes apart. In order to ensure that this sampling procedure did not bias our results, we tested similarity in association patterns between scans taken at 2 (scan 1), 4 (scan 2) and 18 minutes (scan 9) of the focal sample including both sexes. There was no significant difference in the number of times focal and non-focal subjects were in close proximity at scan 1 (Median = 2, IQ range = 0 - 5) and scan 2 (Median = 2, IQ range = 1 – 5, Wilcoxon signed-ranks test, T = 411.50, N = 132, P = 0.435). However, there was a significant difference in the number of times focal and non-focal subjects were in close proximity at scan 1 and scan 9 (Median = 2, IQ range = 1 – 4; Wilcoxon signed-ranks test, T = 2656.50, N = 132, P = 0.011). Similarly, there was no significant difference in the number of times focal and non-focal subjects were in the same party at scan 1 (median = 5, IQ range: 3 - 10) and scan 2 (median = 5, IQ range: 3 - 10; Wilcoxon signed-ranks test, T = 218.50, N = 132, P = 0.571). However, there was a significant difference in the number of times focal and non-focal subjects were in the same party at scan 1 and scan 9 (median = 5, IQ range: 2 - 10; Wilcoxon signed-ranks test, T = 1460, N = 132, P = 0.010). Thus, the adjacent scans were similar for 10 meter associations and party level associations and were treated as continuous. However, first and final sample scans differed for 10 meter associations and party level associations and therefore we established that these scans were independent as well as the samples preceding and succeeding the focal follow.

**SI Table 2.** Categorisation of attributes

| Category of attribute | Subcategory of attribute | Description of subcategory |
| --- | --- | --- |
| Kinship similarity | Kin | mother-son  son-mother |
|  | Non-kin | unrelated dyad |
| Sex similarity | Same sex | male-male  female-female |
|  | Different sex | male-female  female-male |
| Reproductive similarity | Same reproductive state | male-male  male-oestrous female  oestrous female - male  unoestrous female – unoestrous female  oestrous female- oestrous female |
|  | Different reproductive state | unoestrous female-oestrous female  unoestrous female-male |
| Age proximity | Same age category | no more than 5 years age difference between individuals in the dyad |
|  | Different age category | more than 5 years age difference between individuals in the dyad |
| Sex | Male  Female |  |
| Age | Young adult | 16 years old or younger |
|  | Mature adult | More than 16 years of age |

**SI 3. Sampling effort**

To examine the potential influence of the sampling duration on the patterns of recorded behaviour, we used Geary’s C statistic to examine the autocorrelations between the total duration of observation of each focal chimpanzee and four different networks (proximity, grooming, gesture, pant-grunt). There was not a statistically significant relationship between the total duration of observation and the networks for duration of time spent in close proximity (Geary’s *C* = 0.963, *p* = 0.266), grooming duration (C = 0.993, *p* = 0.511), gesture sequence rate (C = 1.011, *p* = 0.436) and pant-grunt rate (C = 0.886, *p* = 0.320). This suggests sufficient sampling duration.

**SI Table 4.** MRQAP regression model showing predictors of duration of time spent in close proximity between N = 12, 132 dyadic relationships of the chimpanzees. Significant P values are indicated in bold.

| Attribute category/ rate of gesture sequence per hour spent in close proximity | Model 1, *r^2^* =0.055 | | |
| --- | --- | --- | --- |
|  | Standardized coefficient | Standard error | *P* |
| Age similarity | 0.139 | 3.734 | 0.096 |
| Sex similarity | -0.073 | 2.999 | 0.235 |
| Kinship similarity | 0.061 | 6.628 | 0.276 |
| Reproductive similarity | -0.018 | 3.205 | 0.400 |
| Gesture sequences combined | 0.184 | 0.287 | **0.027** |

**SI Table 5.** MRQAP regression model showing predictors of duration of time spent in close proximity between N = 12, 132 dyadic relationships of the chimpanzees. Significant P values are indicated in bold.

| Attribute category/ rate of gesture sequence per hour spent in close proximity | Model 2, *r^2^* =0.049 | | |
| --- | --- | --- | --- |
|  | Standardized coefficient | Standard error | *P* |
| Age similarity | 0.140 | 3.677 | 0.087 |
| Sex similarity | -0.061 | 2.989 | 0.281 |
| Kinship similarity | 0.060 | 6.907 | 0.270 |
| Reproductive similarity | 0.003 | 3.226 | 0.523 |
| Threat to dominate | 0.162 | 1.956 | **0.030** |

**SI Table 6.** MRQAP regression model showing predictors of duration of time spent in close proximity between N = 12, 132 dyadic relationships of the chimpanzees. Significant P values are indicated in bold. Definitions of gesture functions are given in Table 2.

| Attribute category/ rate or duration of behaviour per hour spent in close proximity | Model 3, *r^2^* = 0.242 | | |
| --- | --- | --- | --- |
|  | Standardized coefficient | Standard error | *P* |
| Age similarity | 0.199 | 4.043 | **0.021** |
| Sex similarity | -0.075 | 3.377 | 0.234 |
| Kinship similarity | 0.069 | 6.874 | 0.214 |
| Reproductive similarity | -0.065 | 3.015 | 0.245 |
| Threat to dominate | 0.100 | 10.219 | 0.395 |
| Food sharing | -0.010 | 55.956 | 0.441 |
| Other threat | 0.078 | 4.069 | 0.153 |
| Travel | 0.118 | 4.797 | 0.069 |
| Copulation | 0.073 | 1.845 | 0.172 |
| Reassurance | -1.040 | 11.298 | **0.032** |
| Greeting | 0.173 | 1.930 | **0.018** |
| Gesture to mutually groom | 0.908 | 14.582 | **0.048** |
| Gesture to receive groom | 0.082 | 4.044 | 0.350 |
| Gesture to give groom | -0.126 | 1.191 | 0.180 |
| Play | -0.041 | 0.784 | 0.344 |
| Solo high-intensity pant-hoot | 0.064 | 2.983 | 0.202 |
| Synchronized high-intensity pant-hoot | -0.171 | 1.736 | **0.015** |
| Synchronized low-intensity pant-hoot | 0.175 | 4.276 | **0.017** |
| Grooming mutual duration | 0.038 | 0.819 | 0.390 |
| Grooming received duration | 0.215 | 0.769 | **0.017** |
| Grooming given duration | 0.116 | 1.099 | 0.224 |

**SI Table 7.** MRQAP regression model showing predictors of proximity between N = 12, 132 dyadic relationships of the chimpanzees. Significant P values are indicated in bold. Dyads of individuals who had values of proximity association equal or greater than the mean plus half SD, were scored as 1 (‘preferred reciprocated close proximity ties’), if the proximity was reciprocated (i.e. both A to B and B to A displayed values of proximity association equal or greater than the mean plus half SD) whereas dyads who had values less than then mean plus half SD were scored as 0.

| Attribute category/ rate or duration of behaviour per hour spent in close proximity | Model 4, *r^2^* = 0.471 | | |
| --- | --- | --- | --- |
|  | Standardized coefficient | Standard error | *P* |
| Age similarity | 0.176 | 0.100 | **0.039** |
| Sex similarity | 0.001 | 0.082 | 0.470 |
| Kinship similarity | -0.069 | 0.177 | 0.205 |
| Reproductive similarity | -0.213 | 0.090 | **0.018** |
| Threat to dominate | -0.492 | 0.243 | **0.027** |
| Food sharing | -0.031 | 1.415 | 0.317 |
| Other threat | -0.032 | 0.096 | 0.346 |
| Travel | 0.226 | 0.108 | **0.005** |
| Copulation | 0.047 | 0.044 | 0.163 |
| Reassurance | -1.466 | 0.249 | **0.001** |
| Greeting | 0.162 | 0.048 | **0.034** |
| Gesture to mutually groom | 1.579 | 0.324 | **0.001** |
| Gesture to receive groom | 0.707 | 0.090 | **0.001** |
| Gesture to give groom | 0.028 | 0.026 | 0.351 |
| Play | -0.104 | 0.017 | **0.046** |
| Solo high-intensity pant-hoot | 0.017 | 0.073 | 0.321 |
| Synchronized high-intensity pant-hoot | -0.068 | 0.042 | 0.107 |
| Synchronized low-intensity pant-hoot | 0.258 | 0.105 | **0.001** |
| Grooming mutual duration | -0.043 | 0.019 | 0.380 |
| Grooming received duration | 0.069 | 0.018 | 0.185 |
| Grooming given duration | -0.082 | 0.021 | 0.253 |

**SI Table 8.** MRQAP regression model showing predictors of proximity between N = 12, 132 dyadic relationships of the chimpanzees. Significant P values are indicated in bold. Dyads of individuals who had values of proximity association equal or greater than the mean plus half SD, were scored as 1 when the proximity was non-reciprocated (i.e. only A to B but not B to A had duration of proximity association equal or above the 30.3 minutes - ‘preferred, non-reciprocated close proximity bonds’), whereas other dyads were scored as 0.

| Attribute category/ rate or duration of behaviour per hour spent in close proximity | Model 5, *r^2^* = 0.107 | | |
| --- | --- | --- | --- |
|  | Standardized coefficient | Standard error | *P* |
| Age similarity | -0.031 | 0.098 | 0.359 |
| Sex similarity | 0.009 | 0.088 | 0.469 |
| Kinship similarity | 0.078 | 0.171 | 0.166 |
| Reproductive similarity | 0.076 | 0.073 | 0.143 |
| Threat to dominate | 0.857 | 0.326 | 0.065 |
| Food sharing | -0.041 | 1.752 | 0.395 |
| Other threat | 0.027 | 0.119 | 0.224 |
| Travel | -0.060 | 0.140 | 0.220 |
| Copulation | -0.024 | 0.058 | 0.508 |
| Reassurance | -0.553 | 0.340 | 0.189 |
| Greeting | 0.105 | 0.066 | 0.163 |
| Gesture to mutually groom | -0.043 | 0.460 | 0.479 |
| Gesture to receive groom | -0.463 | 0.127 | **0.012** |
| Gesture to give groom | -0.217 | 0.037 | 0.071 |
| Play | 0.010 | 0.025 | 0.361 |
| Solo high-intensity pant-hoot | 0.083 | 0.092 | 0.179 |
| Synchronized high-intensity pant-hoot | -0.114 | 0.050 | **0.024** |
| Synchronized low-intensity pant-hoot | -0.013 | 0.132 | 0.636 |
| Grooming mutual duration | 0.018 | 0.025 | 0.415 |
| Grooming received duration | 0.158 | 0.024 | 0.087 |
| Grooming given duration | 0.209 | 0.035 | 0.116 |

**SI Table 9.** MRQAP regression model showing predictors of proximity between N = 12, 132 dyadic relationships of the chimpanzees. Significant P values are indicated in bold. dyads of individuals who had values of proximity association equal or below the mean minus half SD (who spent 16.23 or less minutes in close proximity to each other per hour spent in same party), were scored as 1 (‘non-preferred close proximity bonds’), whereas other dyads were scored as 0.

| Attribute category/ rate or duration of behaviour per hour spent in close proximity | Model 6, *r^2^* = 0.229 | | |
| --- | --- | --- | --- |
|  | Standardized coefficient | Standard error | *P* |
| Age similarity | -0.202 | 0.128 | **0.013** |
| Sex similarity | 0.057 | 0.106 | 0.274 |
| Kinship similarity | -0.100 | 0.214 | 0.109 |
| Reproductive similarity | 0.021 | 0.089 | 0.400 |
| Threat to dominate | -0.309 | 0.339 | 0.247 |
| Food sharing | -0.054 | 1.568 | 0.323 |
| Other threat | -0.146 | 0.139 | **0.010** |
| Travel | -0.009 | 0.149 | 0.504 |
| Copulation | -0.154 | 0.060 | **0.007** |
| Reassurance | 0.524 | 0.379 | 0.203 |
| Greeting | -0.220 | 0.072 | **0.002** |
| Gesture to mutually groom | -0.172 | 0.492 | 0.381 |
| Gesture to receive groom | 0.150 | 0.139 | 0.224 |
| Gesture to give groom | 0.107 | 0.041 | 0.234 |
| Play | 0.090 | 0.027 | 0.198 |
| Solo high-intensity pant-hoot | -0.035 | 0.100 | 0.362 |
| Synchronized high-intensity pant-hoot | 0.189 | 0.055 | **0.006** |
| Synchronized low-intensity pant-hoot | -0.126 | 0.140 | **0.020** |
| Grooming mutual duration | -0.161 | 0.027 | 0.100 |
| Grooming received duration | -0.204 | 0.026 | **0.002** |
| Grooming given duration | -0.139 | 0.036 | 0.176 |

**SI Table 10.** MRQAP regression model showing predictors of duration of time spent in close proximity between N = 12, 132 dyadic relationships of the chimpanzees. Significant P values are indicated in bold.

| Attribute category/ rate of gesture sequence per hour spent in close proximity | Model 7, *r^2^* =0.066 | | |
| --- | --- | --- | --- |
|  | Standardized coefficient | Standard error | *P* |
| Age similarity | 0.155 | 3.733 | 0.069 |
| Sex similarity | -0.035 | 2.933 | 0.359 |
| Kinship similarity | 0.079 | 6.632 | 0.196 |
| Reproductive similarity | -0.010 | 3.062 | 0.443 |
| Pant-grunt given | 0.209 | 1.511 | **0.010** |

**SI Table 11.** MRQAP regression model showing predictors of pant-grunt given by focal individual between N = 12, 132 dyadic relationships of the chimpanzees. Significant P values are indicated in bold. Description of gesture functions is given in Table 2.

| Attribute category/ rate of communication sequence per hour spent in close proximity | Model 8, *r^2^* =0.807 | | |
| --- | --- | --- | --- |
|  | Standardized coefficient | Standard error | *P* |
| Age similarity | -0.007 | 0.122 | 0.469 |
| Sex similarity | -0.082 | 0.110 | 0.056 |
| Kinship similarity | -0.053 | 0.220 | 0.066 |
| Reproductive similarity | 0.026 | 0.086 | 0.250 |
| Threat to dominate | -0.134 | 0.344 | 0.145 |
| Food sharing | 0.000 | 1.682 | 0.378 |
| Other threat | 0.005 | 0.121 | 0.309 |
| Travel | -0.035 | 0.134 | 0.088 |
| Copulation | 0.000 | 0.056 | 0.623 |
| Reassurance | -0.476 | 0.363 | **0.049** |
| Greeting | 0.925 | 0.209 | **0.001** |
| Gesture to mutually groom | 0.335 | 0.488 | 0.120 |
| Gesture to receive groom | -0.037 | 0.129 | 0.311 |
| Gesture to give groom | 0.013 | 0.038 | 0.372 |
| Play | 0.030 | 0.027 | 0.158 |
| Solo high intensity pant-hoot | -0.001 | 0.102 | 0.570 |
| Synchronized high-intensity pant-hoot | -0.006 | 0.050 | 0.482 |
| Synchronized low-intensity pant-hoot | -0.014 | 0.131 | 0.306 |
| Grooming mutual duration | 0.061 | 0.026 | 0.148 |
| Grooming received duration | 0.019 | 0.024 | 0.190 |
| Grooming given duration | -0.068 | 0.036 | 0.148 |

**SI Table 12.** Node level regression predicting proximity in degree between N = 12 chimpanzees. Significant *P* values are given in bold (Model 9, *r^2^* = 0.422).

| Variable | Standardized coefficient | *P* |
| --- | --- | --- |
| Reproductive state of female | -0.177 | 0.354 |
| Kinship | 0.148 | 0.332 |
| Sex/ age | 0.193 | 0.307 |
| Gesture sequence normalized degree | 0.697 | **0.033** |

**SI Table 13.** Node level regression predicting proximity in degree between N = 12 chimpanzees. Significant *P* values are given in bold (Model 10, *r^2^* = 0.463).

| Variable | Standardized coefficient | *P* |
| --- | --- | --- |
| Reproductive state of female | 0.006 | 0.386 |
| Kinship | 0.255 | 0.243 |
| Sex/ age | -0.016 | 0.503 |
| Pant-grunt given normalized degree | 0.688 | **0.028** |

**SI Table 14.** Node level regression predicting proximity in degree between N = 12 chimpanzees. Significant *P* values are given in bold (Model 11, *r^2^* = 0.908).

| Variable | Standardized coefficient | *P* |
| --- | --- | --- |
| Reproductive state of female | 0.612 | 0.214 |
| Kinship | 0.847 | 0.074 |
| Sex/ age | 1.109 | 0.060 |
| Grooming received normalized degree | 2.830 | **0.047** |
| Reassurance normalized degree | -0.638 | 0.160 |
| Greeting normalized degree | -2.695 | **0.029** |
| Synchronized high-intensity pant-hoot normalized degree | 2.892 | **0.024** |
| Synchronized low-intensity pant-hoot normalized degree | 0.233 | 0.423 |
| Gesture to mutually groom normalized degree | -0.731 | 0.220 |

**SI Table 15.** Node level regression predicting proximity in degree between N = 12 chimpanzees. Significant *P* values are given in bold (Model 12, *r^2^* = 1).

| Variable | Standardized coefficient | *P* |
| --- | --- | --- |
| Reproductive state of female | -2.671 | 0.101 |
| Kinship | 1.085 | 0.183 |
| Sex/ age | 2.052 | **0.048** |
| Greeting degree | -2.848 | 0.105 |
| Gesture to mutually groom normalized degree | -0.627 | 0.407 |
| Synchronized low-intensity pant-hoot normalized degree | 4.994 | **0.031** |
| Travel normalized degree | 1.008 | 0.253 |
| Gesture to receive groom normalized degree | -2.032 | 0.196 |
| Play normalized degree | -0.080 | 0.480 |
| Reassurance normalized degree | -0.469 | 0.387 |
| Threat to dominate normalized degree | 1.978 | 0.235 |
